# Cost-Effectiveness and Cost–Benefit Analyses of Providing Menstrual Cups and Sanitary Pads to Schoolgirls in Rural Kenya

**DOI:** 10.1089/whr.2021.0131

**Published:** 2022-09-15

**Authors:** Masih A. Babagoli, Anja Benshaul-Tolonen, Garazi Zulaika, Elizabeth Nyothach, Clifford Oduor, David Obor, Linda Mason, Emily Kerubo, Isaac Ngere, Kayla F. Laserson, Rhiannon Tudor Edwards, Penelope A. Phillips-Howard

**Affiliations:** ^1^ Department of Economics, Barnard College, Columbia University, New York, USA.; ^2^ Department of Clinical Sciences, Liverpool School of Tropical Medicine, Liverpool, United Kingdom.; ^3^ Centre for Health Research, Kenya Medical Research Institute, Kisumu, Kenya.; ^4^ County Health Headquarters, Ministry of Health, Siaya County, Kenya.; ^5^ Division of Global Health Protection, Center for Global Health, Centers for Disease Control and Prevention, Atlanta, Georgia, USA.; ^6^ Centre for Health Economics and Medicines Evaluations, Bangor University, Bangor, United Kingdom.

**Keywords:** adolescence, cost–benefit analysis, cost-effectiveness analysis, menstrual cup, menstrual health, randomized trial, sanitary pads

## Abstract

**Objective:**

To analyze the relative value of providing menstrual cups and sanitary pads to primary schoolgirls.

**Design:**

Cost-effectiveness and cost–benefit analyses of three-arm single-site open cluster randomized controlled pilot study providing menstrual cups or sanitary pads for 1 year.

**Participants:**

Girls 14–16 years of age enrolled across 30 primary schools in rural western Kenya.

**Methods:**

Cost-effectiveness analysis was conducted based on the health effects (reductions in disability-adjusted life years [DALYs]) and education effects (reductions in school absenteeism) of both interventions. The health and education benefits were separately valued and compared with relative program costs.

**Results:**

Compared with the control group, the cost of menstrual cups was estimated at $3,270 per year for 1000 girls, compared with $24,000 for sanitary pads. The benefit of the menstrual cup program (1.4 DALYs averted, 95% confidence interval [CI]: −4.3 to 3.1) was higher compared with a sanitary pad program (0.48 DALYs averted, 95% CI: −4.2 to 2.3), but the health effects of both interventions were not statistically significant likely due to the limited statistical power. Using point estimates, the menstrual cup intervention was cost-effective in improving health outcomes ($2,300/DALY averted). The sanitary pad intervention had a cost-effectiveness of $300/student-school year in reducing school absenteeism. When considering improvements in future earnings from reduced absenteeism, the sanitary pad program had a net benefit of +$68,000 (95% CI: −$32,000 to +$169,000).

**Conclusions:**

The menstrual cup may provide a cost-effective solution for menstrual hygiene management in low-income settings. This study outlines a methodology for future analyses of menstrual hygiene interventions and highlights several knowledge gaps that need to be addressed. Trial registration: ISRCTN17486946.

## Introduction

The context surrounding menstrual health management (MHM) among adolescent girls—which includes sociocultural norms, access to MHM products and sanitary facilities, and knowledge about menstrual health—impacts the incidence of sexual and reproductive health harms, school attendance and performance, and psychosocial health, all of which can limit girls from reaching their full potential.^1–10^ Providing MHM interventions, such as menstrual cups and sanitary pads, could mitigate these adverse effects. This raises questions as to the relative benefits and cost-effectiveness of various MHM interventions.

Until recently, reusable menstrual cups have received little attention and have not been widely promoted in comparison with single-use products such as sanitary pads; for example, a recent review of puberty and menarche educational materials across 27 countries found that only 30% mentioned menstrual cups, compared with 77% for disposable pads.^11^ Evidence indicates menstrual cups are a good option for MHM.^11^ Menstrual cups are durable and can last several years, although there is a higher up-front cost when compared with other MHM products such as sanitary pads, which some non-governmental organizations and governments provide to school-age girls.^12^ For example, the Kenyan government has a program that provides sanitary pads for free to public secondary schools across the country, although there have been various challenges in program implementation.^13,14^

Regardless, no comprehensive economic analysis to inform large-scale MHM programming and compare the relative impacts of different MHM interventions has been undertaken. Cost–benefit analyses (CBA) and cost-effectiveness analyses (CEA) are warranted to push effective national and international policies and scale-up of MHM programming.

We explore the costs and benefits associated with a randomized controlled feasibility study that analyzed the health and education impacts of sanitary pads and menstrual cups targeted to primary school girls in Siaya County in western Kenya. Previously reported results of the feasibility study provide the backdrop for this CEA and CBA.^4,10,15–18^ The feasibility study found reductions in the incidence of some sexually transmitted infections (STIs) and reproductive tract infections (RTIs) among participants provided MHM products, as well as reductions in school absenteeism among those provided sanitary pads.^4,10^

## Methods

### Parent study

The interventions in the feasibility study were randomly allocated at the school level and comprised three arms: (1) sanitary pads (Always^®^ brand sourced from local stores), (2) a menstrual cup (Mooncup^®^ brand sourced from the United Kingdom), or (3) business as usual (control). Thirty schools (10 per arm) in Siaya County, Western Kenya, participated in the study. Girls who were 14–16 years old, had experienced at least three menses, and had no disability precluding their ability to participate were invited to partake in the study.

For ethical reasons, all students in the program, including those in the control group, received soap and puberty training, and all schools received soap for their handwashing stations. No further investments were undertaken regarding water and sanitation facilities.^4^ The feasibility study was funded by the UK Medical Research Institute, Department for International Development, and Wellcome Trust Global Health Trials. The results from health outcomes measured in the feasibility study are summarized in Table S1 in Supplementary Data.

### Program costs

Based on the program costs for the feasibility study, the cost of the control group (provision of puberty education and soap) and the cost of providing menstrual cups or sanitary pads for 1000 individuals for 1 year was calculated. Program costs were calculated from the perspective of a government program or health care payer providing these interventions, including the necessary logistics and personnel for such a program.^19^ Local bulk order prices were used for the MHM products. Sixteen sanitary pads (two packs) were estimated to be required per month, representing an upper bound estimate. This is based on the estimated need of each girl assuming a regular period lasting 4 to 5 days with four changes in the heavier 3 days and two changes in the lighter 2 days. This estimate may not align with current government programs that supply fewer pads due to logistical or budgetary constraints.

One reusable silicone menstrual cup is predicted to last 10 years. Therefore, one-tenth of the cost for a menstrual cup was considered since program costs were calculated for 1 year. This assumption is consistent with a recent review that accumulated cost estimates over 10 years to compare the financial and environmental costs of menstrual cups and pads.^11^ Given the menstrual cup may have to be replaced if lost, a menstrual cup replacement cost was calculated using the proportion of menstrual cups lost in the feasibility study.^17^ As seen in other menstrual cup programs and studies,^20–22^ individuals receiving menstrual cups require additional training/supervision to ensure proper use and care, and costs for this training reflect those incurred during the feasibility study. The feasibility study also informs the unit costs for the remaining costs not aforementioned.

Research administrative costs were not included to make costs align with potential future large-scale programs. Private costs were not included. Costs were considered over a 1-year period, so no time discounting was required. Lastly, for all currency conversions between Kenya Shilling (KES) and United States Dollar (USD), a conversion rate equating 1 USD to 101 KES was used, reflecting the exchange rate at the time of writing.^23^

Lastly, while the unit prices for menstrual cups and sanitary pads from the feasibility study were used in the main analysis, a sensitivity analysis was conducted considering a range of costs for menstrual cups and sanitary pads. For menstrual cups, the price range included an upper bound, which reflected the highest price for a menstrual cup in Africa.^11^ For sanitary pads, the price range reflected the cost of different quality brands. More details are available in Tables S12 and S13 in Supplementary Data.

### Health effects

The randomized feasibility study measured the effect of providing menstrual cups or sanitary pads on the incidence of chlamydia, gonorrhea, trichomoniasis, bacterial vaginosis, and candidiasis.^4^ In addition to causing acute symptomatic infections, these STIs and RTIs in females can result in long-term sequelae, including pelvic inflammatory disease, ectopic pregnancy, and tubal infertility,^24^ and can facilitate acquisition and transmission of HIV infection.^25,26^ Disability-adjusted life years (DALYs) were used to measure the effects of the interventions in decreasing disease burden, including both the initial infection and the future sequelae from chronic infection.

Zero DALYs indicate a year lived in perfect health, one indicates death, and an intermediate value is the equivalent of a year of life lived in less than perfect health.^27,28^ To calculate DALYs, the years of life lost and years lost to disease were summed. We did not apply either age weighting or time discounting. We assumed a life expectancy of 86 years, consistent with Global Burden of Diseases studies.^29^ Full details of health effects calculation for all five outcomes are included in the Supplementary Data.

The intervention in this feasibility study resulted in a statistically significant reduction in prevalence of overall STIs comparing the menstrual cup and control arms. However, when looking at individual STIs and RTIs, there was only a statistically significant reduction in incidence of chlamydia (Table S1 in Supplementary Data).^4^ Nonetheless, since the feasibility study had limited statistical power and this analysis aimed to create a framework for future MHM economic analyses, all five measured health outcomes were considered, and the DALYs for all health outcomes were summed. Additionally, the median length of follow-up of study participants in the feasibility study was 10.9 months.^4^ The incidence of health outcomes was extrapolated to 12 months of intervention, assuming a linear relationship between intervention duration and incidence.

To conduct a CEA, the total cost of an intervention program providing either menstrual cups or sanitary pads to 1000 individuals for 1 year was considered relative to the cost of the control group. To calculate the incremental cost-effectiveness ratio (ICER), the relative cost was divided by the number of DALYs that would be averted through such a program. The ICER was compared with cost-effectiveness thresholds suggested by the World Health Organization (WHO).^30^

Lastly, to conduct a CBA, DALYs averted by each intervention were valued monetarily using a willingness-to-pay (WTP) approach^19,31,32^ and then compared with the cost of that intervention relative to the control group. We used a WTP method previously described^33^ to value a quality-adjusted life year. To estimate the value of an averted DALY, the value of a statistical life (VSL) was divided by the average residual life expectancy of the population for which the VSL was estimated. The VSL used for Kenya was $230,000, estimated by Viscusi and Masterman^34^ using a base US VSL of $9.6 million and then adjusting that value using the relative Gross National Income and income elasticity in Kenya.

### Education effects

A previous analysis showed that providing menstrual cups or sanitary pads to school-age girls had no statistically significant effect on school dropout rates.^4^ Menstrual cups also had no effect on school absenteeism; however, sanitary pads led to a 7.9% (95% confidence interval [CI]: −0.72% to 17%) reduction in absenteeism.^10^ In other words, provision of sanitary pads to 1000 school-age girls for 1 year leads to an increase in attendance of 79 (95% CI: −7.2 to 170) student school years. To evaluate the ICER of sanitary pads in improving school attendance, the relative cost of a program providing sanitary pads to 1000 school-age girls for 1 year was divided by the increase in years of schooling due to the intervention.

To conduct a CBA, a human capital approach was adopted to monetize the benefit of an increase in school attendance and compare the benefit to the relative cost of the intervention.^35^ The rate of return to education in Kenya is estimated at 17%,^36^ which we assumed was applicable to any increment of a year. That rate of return is decomposed to a 7% return on each additional year of primary school.^35^ Consistent with a previous analysis, we assumed that each individual will earn wages for 40 years and that wages are 60% of the output per worker on average.^35^ To calculate the net present value of the increase in future wages, a discount rate of 5% was applied.

### Ethics approval

The randomized feasibility study obtained ethics approval from the Kenyan Scientific and Ethics Unit (#2198) and the Research and Ethics Committee of the Liverpool School of Tropical Medicine (#12-11). Verbal consent and assent were collected from the head teachers and participating girls before the study, and written informed consent was collected from the parents, as outlined in the feasibility studies.^4,10^

## Results

### Program costs

Based on the perspective of a government/health care program, the annual program cost for the baseline (control) scenario, including puberty education and soap, was $3.44 per student (Table 1). In comparison, the annual program costs for the provision of menstrual cups and sanitary pads were $6.71 and $27.44, respectively (Table 1). Private and environmental costs—such as the cost of usual practice in the control group, firewood and water for boiling in the menstrual cup group, and disposal in the sanitary pad group—were not considered. Relative to the control group, provision of menstrual cups costs $3.27 annually per student, and provision of sanitary pads costs $24.00 annually per student (Table 1).

**Table 1.** Program Costs for Each Treatment Arm of Study

	Treatment arm	(1) Control^a^		(2) Menstrual cups		(3) Sanitary pads	
	Item	Annual cost per student (USD)	Notes/assumptions	Annual cost per student (USD)	Notes/assumptions	Annual cost per student (USD)	Notes/assumptions
Material costs	Sanitary pads					24	1 USD/pack; 2 packs/month
	Menstrual cups			1.00	Total cost 10 USD; single cup lasts 10 years		
	Replacement for lost menstrual cups			0.05	6.3% of students lost their menstrual cups (van Eijk et al^17^)		
Menstrual cup training	Repeat training for students by nurses			1.53	Two half-day class repeat trainings required annually; 22 students on average per class		
	Training for girls, menstrual cup usage			0.19	1 hour of class training in addition to puberty education		
	Training materials, menstrual cup usage			0.50			
Control costs (puberty education and hygiene)	Soap for hygiene	1.50	1 soap required per term; total 3 terms per year	1.50	1 soap required per term; total 3 terms per year	1.50	1 soap required per term; total 3 terms per year
	Training for nurses	0.17	3 hours training required; each nurse trains 75 students on average	0.17	3 hours training required; each nurse trains 75 students on average	0.17	3 hours training required; each nurse trains 75 students on average
	Training for girls, puberty education	0.77	2 hours of class training plus travel time; 22 students on average per class	0.77	2 hours of class training plus travel time; 22 students on average per class	0.77	2 hours of class training plus travel time; 22 students on average per class
	Training materials, puberty education	1.00		1.00		1.00	
		**Total 3.44 USD**		**Total 6.71 USD**		**Total 27.44 USD**	
				**Relative to control 3.27 USD** ^b^		**Relative to control 24.00 USD** ^b^	

Costs are considered from the perspective of a government/health care program, excluding private costs.

^a^ The private cost of usual practice varies based on individual practices.

^b^ The cost of each intervention relative to the control arm was used in cost-effectiveness and cost–benefit analyses since the health and education effects of each intervention were also measured in comparison to the control arm.

USD, United States Dollar.

### Health effects

Prevalence of each infection was linearly extrapolated to a study period of 12 months (Supplementary Data). The total DALYs per infection, including the long-term sequalae, of chlamydia and gonorrhea were calculated to be 0.0081 and 0.033 years, respectively (Table 2, column 2). DALYs per case of trichomoniasis was 0.012 years, bacterial vaginosis was 0.011 years, and candidiasis was 0.011 years (Table 2, column 2) (calculations detailed in Supplementary Data).

**Table 2.** Health Effects and Value of Reductions in Infections Due to Menstrual Health Management Intervention

		(3) Reductions in infections/1000 provided intervention^b^		(4) DALYs averted/1000 provided intervention		(5) Valuation of DALYs averted/1000 provided intervention	
(1) Pathogen (infection)	(2) DALYs/infection^a^	Cups vs. control	Pads vs. control	Cups vs. control	Pads vs. control	Cups vs. control	Pads vs. control
*Chlamydia trachomatis* (chlamydia)	0.0081	27 (−24 to 43)	33 (6.9 to 44)	0.22 (−0.20 to 0.34)	0.27 (0.056 to 0.35)	$1,100 (−$960 to $1,700)	$1,300 ($270 to $1,700)
*Trichomonas vaginalis* (trichomoniasis)	0.012^c^	32 (−14 to 44)	17 (−51 to 39)	0.40 (−0.17 to 0.55)	0.22 (−0.63 to 0.49)	$1,900 (−$850 to $2,700)	$1,100 (−$3,100 to $2,400)
*Neisseria gonorrhea* (gonorrhea)	0.033	−0.46 (−70 to 5.9)	1.5 (−41 to 6.1)	−0.015 (−2.3 to 0.20)	0.051 (−1.4 to 0.20)	−$76 (−$11,000 to $970)	$250 (−$6,700 to $990)
Bacterial vaginosis	0.011^c^	65 (−18 to 120)	6.8 (−99 to 79)	0.75 (−0.21 to 1.4)	0.077 (−1.1 to 0.90)	$3,700 (−$1,000 to $6,700)	$380 (−$5,600 to $4,400)
*Candida albicans* (candidiasis)	0.011^c^	7.3 (−130 to 59)	−13 (−100 to 36)	0.079 (−1.4 to 0.64)	−0.14 (−1.1 to 0.39)	$390 (−$6,900 to $3,100)	−$680 (−$5,500 to $1,900)
Total				1.4 (−4.3 to 3.1)	0.48 (−4.2 to 2.3)	$6,900 (−$21,000 to $15,000)	$2,400 (−$21,000 to $11,000)

Values in parentheses include 95% confidence intervals.

^a^ Infection refers to both symptomatic and asymptomatic infections.

^b^ Calculated using prevalence ratios from Table S1 in Supplementary Data.

^c^ The DALYs/infection for *T. vaginalis*, bacterial vaginosis, and *C. albicans* only consider the increased risk of contracting chlamydia, gonorrhea, and/or HIV as a result of the initial infection. This is in contrast to DALYs/infection for *C. trachomatis* and *N. gonorrhea* that include all potential long-term effects of the infections.

DALYs, disability-adjusted life years.

Considering the prevalence of each infection in the control group and the prevalence ratio of infections in the treatment groups, the reductions in each infection and DALYs averted were calculated for a potential program providing menstrual cups or sanitary pads to 1000 individuals for 1 year (Table 2, columns 3–4). For a program providing menstrual cups, the greatest reduction in infections and DALYs averted was for bacterial vaginosis, 65 fewer infections per 1000 individuals provided intervention and 0.75 DALYS averted. For a program providing sanitary pads, the greatest effect was for chlamydia, 33 fewer infections per 1000 individuals provided intervention and 0.27 DALYs averted. Overall, provision of menstrual cups to 1000 individuals was estimated to avert 1.4 DALYs (95% CI: −4.3 to 3.1), whereas provision of sanitary pads was estimated to avert 0.48 DALYs (95% CI: −4.2 to 2.3) (Table 2, column 4).

#### CEA of health effects

Considering that the relative cost of providing menstrual cups to 1000 participants in Kenya for 1 year would be $3,270 (Table 1), the point estimate for the ICER of this intervention was $2,300 per DALY averted (95% CI: $1,100 to dominated) (Table 4, column 2b). Analogously, the relative cost of providing sanitary pads to 1000 school-age girls in Kenya would be $24,000 (Table 1); therefore, the point estimate for the ICER of this intervention was $50,000 per DALY averted (95% CI: $10,000 to dominated) (Table 4, column 3b).

**Table 4.** Summary of Costs and Benefits (United States Dollar) of Providing Menstrual Cups or Sanitary Pads to 1000 School-Age Girls for 1 Year

	(1) Relative program cost	(2) Relative health effects				(3) Relative education effects		
		(a) DALYs averted	(b) CEA (USD/DALY averted)	(c) Valuation of averted DALYs	(d) CBA	(a) CEA (USD/student-school year)	(b) Valuation of increased student-school years	(c) CBA
Menstrual cups program (1000 individuals)	$3,270	1.4 (−4.3 to 3.1)	$2,300/DALY averted ($1,100 to dominated)	$6,900 (−$21,000 to $15,000)	Net: +$3,630 (−$24,270 to +$11,730)	No significant effects of menstrual cup provision on absenteeism	No significant effects of menstrual cup provision on absenteeism	Net: −$3,270
Sanitary pads program (1000 individuals)	$24,000	0.48 (−4.2 to 2.3)	$50,000/DALY averted ($10,000 to dominated)	$2,400 (−$21,000 to $11,000)	Net: −$21,600 (−$45,000 to −$13,000)	$300/student-school year ($100 to dominated)^a^	$92,000 (−$8,000 to $193,000)	Net: +$68,000 (−$32,000 to +169,000)

Values in parentheses include 95% confidence intervals.

^a^ Based on education impacts of sanitary pad program reported by Benshaul-Tolonen et al.^10^

CBA, cost–benefit analyses; CEA, cost-effectiveness analyses.

#### CBA based on health effects

Given that Viscusi and Masterman^34^ estimated a VSL of $230,000 in Kenya, a median age in Kenya of 20 years,^37^ and a life expectancy of 67 years,^38^ 47 DALYs were valued at $230,000 in Kenya. Therefore, each DALY was estimated to be $4,900.

Given that provision of menstrual cups to school-age girls was calculated to result in 1.4 DALYs averted (95% CI: −4.3 to 3.1) per 1000 individuals treated, the benefit of the health effects would be $6,900 per year (95% CI: −$21,000 to $15,000) (Table 2, column 5). Comparing the monetized health benefits of menstrual cups to the relative intervention costs, the net effect was estimated to be +$3,630 (95% CI: −$24,270 to +$11,730) (Table 4, column 2d). A similar program providing sanitary pads to school-age girls would result in 0.48 DALYs averted (95% CI: −4.2 to 2.3) per 1000 individuals treated, valued at $2,400 per year (95% CI: −$21,000 to $1,100) (Table 2, column 5). Comparing the benefits to relative intervention costs, the net effect was estimated to be −$21,600 (95% CI: −$45,000 to −$13,000) (Table 2, column 2d).

### Education effects

#### Cost-effectiveness analysis

Given relative program costs of $24,000 (Table 1) to provide sanitary pads to 1000 school-age girls for 1 year, the ICER of a sanitary pad intervention was $300/student school year (95$ CI: $100 to dominated) (Table 4). Menstrual cups did not improve school attendance.^10^

#### CBA based on education effects

Assuming no wage growth and wages as 60% of Gross Domestic Product per capita on average, the annual wage of an individual in Kenya was estimated to be $957. Using a 7% return for each additional year of schooling, the result of the average 0.079 years of schooling per individual gained with the sanitary pad intervention was a $5 increase in the annual wage to $962 per year (Table 3, column 2). As calculated according to the human capital approach, the increase in the net present value of wages over the course of 40 years (discounted 5% annually) resulting from 1-year provision of sanitary pads was, on average, $92 per individual (Table 3, column 4).^†^ Therefore, the monetized benefit of a program providing sanitary pads to 1000 school-age girls for 1 year was estimated to be $92,000 (95% CI: −$8,000 to $193,000) (Table 4, column 3b). Comparing the monetized education benefits with the relative intervention cost, the net effect of providing sanitary pads to 1000 individuals was +$68,000 (95% CI: −$32,000 to $169,000) (Table 4, column 3c).

**Table 3.** Effect of Sanitary Pad Program on Earnings as a Result of Decreased Absenteeism

(1) Group	(2) Annual wage per individual	(3) Total wages over 40 years per individual, discounted at 5% per year	(4) Difference in NPV of wages per individual
No intervention	$957	$16,680	+$92 (−$8 to $193)
Sanitary pad intervention	$962 ($957 to $968)	$16,773 ($16,672 to $16,873)	

Based on education impacts of sanitary pad program reported by Benshaul-Tolonen et al.^10^ Values in parentheses include 95% confidence intervals.

NPV.

## Discussion

### Review of main findings

Overall, the findings from this analysis suggest that providing menstrual cups is less costly than providing sanitary pads, provision of menstrual cups may be more cost-effective than sanitary pads in improving health outcomes, and sanitary pads are possibly cost saving due to their positive impacts on school absenteeism. Specifically, the provision of menstrual cups to 1000 individuals was less costly than the provision of sanitary pads ($3,270 vs. $24,000), in part, due to the 10-year lifespan of the menstrual cups.

The health impacts of the menstrual cup intervention, in terms of DALYs averted due to reductions in STIs and RTIs, were greater compared with the health impacts of the sanitary pad intervention, but the difference was not statistically significant. Estimation of the health impacts and the resulting ICERs was not precise likely due to the limited power of the feasibility study. However, using the point estimates of the health benefits, the ICER of menstrual cups was $2,300 per DALY averted, and the ICER of sanitary pads was $50,000 per DALY averted, suggesting that the provision of menstrual cups may be more cost-effective than the provision of sanitary pads.

WHO's Commission on Macroeconomics and Health suggested that an intervention is cost-effective up to a value three times GDP per capita, which would be $4,785 in Kenya.^30^ Thus, the ICER of a menstrual cup program would be approximately one-half of WHO's recommended value and cost-effective. The ICER of a sanitary pad program would be 10 times the WHO-recommended value. To put these estimates into perspective, menstrual cups are of similar cost-effectiveness for adolescent girls as distributing cholera and typhoid vaccines and treating obstructed labor with cesarean delivery as well as interventions focused on constructing piped water supply and sewer connections to improve sanitation in rural areas.^39^

When exploring effects of the MHM interventions on school attendance, the ICER of the sanitary pad intervention was $300/student-school year using the point estimate of the education benefits. For comparison, the cost-effectiveness of a mass deworming campaign is estimated to be $3.50/additional year of school participation.^35^ The sanitary pad treatment arm has comparable effect on school absenteeism per dollar spent to programs focusing on providing merit scholarships for girls in Kenya, but it has better value than providing conditional cash transfers to promote attendance among girls.^40^ Additionally, when considering the net present value of increased future earnings arising from reduced school absenteeism, sanitary pads are possibly cost saving, although the finding was not statistically significant.

Many CEAs and CBAs focus on interventions that impact a single health outcome or, at least, only impact health-related outcomes. In this case, we attempt to integrate multiple health and education benefits—including STIs, RTIs, school dropout, and school absenteeism—of MHM interventions. However, MHM not only may improve health outcomes outside of the five STIs/RTIs measured in the pilot study but also may have benefits outside of solely health or education (discussed below). Therefore, our analysis is not meant to be an exhaustive account of all possible MHM benefits nor is it intended to argue against MHM provision. Rather, it is meant to provide some evidence of the relative efficiency of different interventions in addressing the need for improved MHM, which we consider a necessity and right for all females.

The methodology outlined in this analysis, including the range of outcomes and costs included, can provide a basis for future studies to capture long-term health impacts as well as nonhealth impacts of MHM interventions. Additionally, our analysis highlights knowledge gaps and methodological challenges that require attention to allow future analyses to evaluate interventions with benefits across multiple areas.

### Limitations and considerations for future studies

The cost and benefit analyses are based on previously published results from a randomized feasibility study. Below we note several challenges in calculating and integrating the impacts of the intervention. First, improving MHM may have health effects beyond the five STIs and RTIs diagnosed in the parent study. To conduct CBA, the health effects (DALYs averted) were monetized using a WTP approach and the education effects (reduction in absenteeism) using a human capital approach; each valuation was separately compared with relative intervention costs. While a WTP approach theoretically includes all three categories of health benefits (direct savings from averted health care expenditures, indirect savings from restored earnings, and intangible savings from averted pain and suffering), a human capital approach only considers improvements in future earnings.^32^ This implies that DALYs averted from each intervention in this study should subsume all benefits arising from each MHM method.

However, in our analysis of sanitary pads, the educational benefits monetized using a human capital approach ($92,000) were undoubtedly larger than the reductions in DALYs monetized using a WTP approach ($2,400), suggesting that health outcomes measured in this feasibility study and converted to DALY reductions did not encompass all of the benefits of the MHM intervention. Therefore, it is possible that there were health benefits of MHM provision that were not accounted for in the study, such as HIV, psychosocial wellbeing, and pregnancy. Additionally, we encourage further studies to monitor effects on student performance, future wages, and productivity (Table S11 in Supplementary Data). Lastly, the effects of the MHM interventions on stigma were not measured in feasibility study and, therefore, not included in our DALY estimate.

Common narratives of fears and stigmas surrounding menstruation found in qualitative^2,5,6^ and quantitative^41^ studies make it imperative that future studies also measure and value the improvements in stigma.^42^ For example, in focus group meetings after 6 months of program implementation, girls participating in the treatment arms of the study reported not being hindered by embarrassment, leakage, and odor and could participate more freely in education—including increased concentration—in contrast to girls in the control group.^20^ Some of these aspects were not captured in the analysis due to lack of quantifiable data but should nonetheless be considered when evaluating menstrual hygiene policies.

Second, we could not identify any disease progression models for trichomoniasis, bacterial vaginosis, and candidiasis to account for long-term sequalae that may result from these infections. Instead, we only considered the increased risks for contracting other infections; for example, bacterial vaginosis has been associated with an increased risk of HIV, chlamydia, and gonorrhea.^43–45^ Regardless, the longer term health impact of these outcomes was underestimated in our analysis. Given that lack of appropriate MHM is associated with increased prevalence of bacterial vaginosis, the long-term impacts of such infections need to be further studied and mapped for future analyses to accurately account for all sequelae.^4,46^

Third, the transition probabilities between health states and the duration of each health state were taken from a European model (Burden of Communicable Diseases in Europe toolkit) since a comprehensive study providing transition probabilities and durations in rural Kenya was not found. These DALY parameters likely underestimate the benefits of infections averted due to differences in access to screening, prognosis, and curative health care between the European and Kenyan populations.

STIs, such as chlamydia, have high costs in terms of morbidity and mortality in developing countries with low access to diagnostic tools.^47^ Notably, a previous study considering the burden of STIs, including downstream cases, in a sexually active population of commercial sex workers in sub-Saharan Africa estimated 5.08 DALYs averted from each treated gonorrhea or chlamydia case. This is significantly higher than our calculation.^47^ Using this DALY estimate in our evaluation, both menstrual cups and pads would be cost-effective at $24/DALY averted and $18/DALY averted, respectively, and cost-saving with net benefits of $670,000 and $820,000, when considering solely the benefits from reductions in chlamydia.

Fourth, the menstrual cup adoption rate accelerated over time, such that treatment effects may be significantly underestimated in the short time frame. Individuals with >9 months of follow-up showed significantly higher health benefits than those with fewer months of follow-up.^4^ However, due to the small sample sizes in this feasibility study, we calculated the prevalence estimates with all study participants and linearly scaled the estimates to a hypothetical 1-year of intervention. Long-run adoption rates and treatment effects should be considered in future trials with longer interventions.

Fifth, three main positive externalities were not included: (1) impact of improved menstrual hygiene on the household members; (2) long-term reduction in infection rates among nonstudy participants due to a lower disease transmission in the population; and (3) lower waste and environmental costs of menstrual cups compared with sanitary pads or cloth. A menstrual cup leads to 0.01 kg of waste is per person per year^48^ compared with 1.3–2.7 kg of waste when sanitary pads are used.^11^ Additionally, disposing of used sanitary pads can cause psychological stress if disposal mechanisms are lacking^2^ and lead to environmental contamination or high pressure on latrines. Moreover, private costs of MHM, such as obtaining water to wash the MHM products, are not considered in this analysis due to a lack of data. We encourage future analyses of menstrual health interventions to measure and consider the private costs and externalities associated with different MHM methods.

Sixth, CEAs typically offset the cost of providing an intervention by health care savings brought about by the intervention to derive the net cost of the intervention. While this pilot study provided treatment to participants who acquired an STI or RTI, it was observed that most girls in the setting did not seek health care for similar causes. Furthermore, for those that did seek care, most of the health care costs were borne by the families of the girls, not by a public payer. Given that this CEA analysis was conducted from a health care payer perspective, we did not adjust for potential health care savings stemming from averted infections. Future analyses should collect data on the private costs of health care utilization and conduct a CEA from a societal perspective.

Lastly, it is fair to assume less than universal uptake of the menstrual cup; while 96% of study participants verbally reported using the menstrual cup after 9 months of enrollment, a usage rate of 70.8% of menstrual cups was color verified.^17^ These less-than-perfect adoption rates are already considered in the estimation of the health and education benefits, as they rely on intent to treat, considering the program effects on the individuals that received the menstrual cup regardless of whether they used them.

## Conclusions

This analysis provides evidence that interventions to improve MHM may have cost-effective benefits on health and education. Menstrual cups could be provided at a significantly lower cost than sanitary pads. We outline a methodology for future CEA and CBA to integrate the multiple effects of menstrual hygiene interventions. While this study does not provide a definitive conclusion, it does lay the framework for future analyses of MHM evaluations and elucidates the current gaps in knowledge and methodological challenges that need to be addressed before other similar analyses can be robustly conducted.

## Supplementary Material

Supplemental data

## Data Sharing Statement

No primary data were analyzed in this article.

## Abbreviations Used

CBA: cost–benefit analyses
CEA: cost-effectiveness analyses
CI: confidence interval
DALYs: disability-adjusted life years
ICER: incremental cost-effectiveness ratio
KES: Kenya Shilling
MHM: menstrual health management
RTIs: reproductive tract infections
STIs: sexually transmitted infections
USD: United States Dollar
VSL: value of a statistical life
WHO: World Health Organization
WTP: willingness-to-pay

## Notes

^†^
Given that many girls do not attend secondary school and enter the workforce after completing primary school, it is justified to assume that the 40 years of wage earning will begin immediately after the intervention. However, if we assume a 4-year delay into the workforce, the increase in net present value of wages over the course of 40 years is $75 per individual.
