# Supplementary material for: Cost-Effectiveness and Cost–Benefit Analyses of Providing Menstrual Cups and Sanitary Pads to Schoolgirls in Rural Kenya
Source: Womens Health Rep (New Rochelle). 2022 Sep 15;3(1):773–84. doi: 10.1089/whr.2021.0131 (PMC9518800; doi:10.1089/whr.2021.0131)
Supplement: Supplemental data [file Suppl_Data.docx]

**Cos-effectiveness and cost-benefit analyses of providing menstrual cups and sanitary pads to schoolgirls in rural Kenya**

Masih A Babagoli^1^*, Anja Benshaul-Tolonen^1^*^†^, Garazi Zulaika^2^, Elizabeth Nyothach^3^, Clifford Oduor^3^, David Obor^3^, Linda Mason^2^, Emily Kerubo^3^, Isaac Ngere^4^, Kayla F. Laserson, Rhiannon Tudor Edwards^5^, Penelope A Phillips-Howard^2^

^1^ Barnard College, Columbia University, USA

^2^ Liverpool School of Tropical Medicine, UK

^3^ Kenya Medical Research Institute, Kenya

^4^ Ministry of Health, Siaya County, Kenya

^5^ Bangor University, UK

*These authors contributed equally to the manuscript.

^†^Corresponding author: atolonen@barnard.edu

Supplementary Information

I. Prevalence of health endpoints

Table S1: Prevalence of health endpoints by intervention group extrapolated to 12 months of intervention.

II. Calculation of disability-adjusted life years

III. Chlamydia (*C. trachomatis*) health impact calculation

Figure S1: Pathogen-based disease model for chlamydia.

Table S2: Inputs used in calculating DALYs per chlamydia infection and associated long-term sequelae.

Table S3: DALY calculation per chlamydia infection and associated long-term sequelae.

IV. Gonorrhea (*N. gonorrhea*) health impact calculation

Figure S2: Pathogen-based disease model for gonorrhea.

Table S4: Inputs used in calculating DALYs per gonorrhea infection and associated long-term sequelae.

Table S5: DALY calculation per gonorrhea infection and associated long-term sequelae.

V. HIV health impact calculation (*for use in health impact calculation for other infections)

Figure S3: Pathogen-based disease model for HIV.

Table S6: Inputs used in calculating DALYs per HIV infection and associated long-term sequelae.

Table S7: DALY calculation per HIV infection and associated long-term sequelae.

VI. Bacterial vaginosis (BV) health impact calculation

Table S8: DALY calculation per bacterial vaginosis infection as a result of increased risk for other health states.

VII. Trichomoniasis (*T. vaginalis*) health impact calculation

Table S9: DALY calculation per trichomoniasis infection as a result of increased risk for other health states.

VIII. *C. albicans* infection health impact calculation

Table S10: DALY calculation per trichomoniasis infection as a result of increased risk for other health states.

IX. Costs and outcomes overview

Table S11: Factors to consider in CB and CE analysis of menstrual hygiene interventions

X. Trial flow diagram

Figure S4: Flow diagram of study participants

XI. CEA/CBA sensitivity analyses based on range of product costs

Table S12: Sensitivity analysis of program costs for each treatment arm of study. Costs are considered from the perspective of a government/healthcare program, excluding private costs. Values in brackets indicate range considered for sensitivity analysis.

Table S13: Sensitivity analysis of costs and benefits (USD) for providing menstrual cups or sanitary pads to 1,000 school-age girls for one year.

I. Prevalence of health endpoints

**Table S1: Prevalence of health endpoints by intervention group extrapolated to 12 months of intervention.**

| (1) Pathogen (infection) | (2) Intervention group | (3) Scaled prevalence of infection | (4) Prevalence ratio^†^  (95% CI) | (5) P-value^†^ |
| --- | --- | --- | --- | --- |
| *C. trachomatis* (chlamydia) | cups | 2.3% | 0.46 (0.14, 1.49) | .195 |
|  | pads | 1.6% | 0.33 (0.12, 0.86) | .024 |
|  | control | 5.0% |  |  |
| *T. vaginalis* (trichomoniasis) | cups | 1.8% | 0.36 (0.11, 1.28) | .115 |
|  | pads | 2.5% | 0.65 (0.21, 2.02) | .457 |
|  | control | 5.0% |  |  |
| *N. gonorrhea* (gonorrhea) | cups | 0.71% | 1.07 (0.10, 11.56) | .956 |
|  | pads | 0.51% | 0.77 (0.08, 7.24) | .816 |
|  | control | 0.66% |  |  |
| Bacterial vaginosis | cups | 16% | 0.71 (0.47, 1.08) | .110 |
|  | pads | 22% | 0.97 (0.65, 1.44) | .864 |
|  | control | 23% |  |  |
| *C. albicans*  (candidiasis) | cups | 8.4% | 0.92 (0.35, 2.43) | .871 |
|  | pads | 10% | 1.14 (0.61, 2.13) | .682 |
|  | control | 9.1% |  |  |

^†^Adopted from Phillips-Howard et al. 2016

II. Calculation of disability-adjusted life years

In the feasibility study, the health effect of the MHM interventions was based on the reductions in specific acute infections – chlamydia, trichomoniasis, gonorrhea, bacterial vaginosis, and candidiasis (Phillips-Howard et al, 2016). However, each of the acute infections can transition to new and more long-term health state with different disability weights and durations and/or can increase an individual’s risk of acquiring other infections. We follow the progression of health states that can potentially result from each pathogen in order to account for the overall disease burden associated with each prevented case of an infection (Devleesschauwer et al. 2014; Mangen et al. 2013). This approach has been previously utilized in the Burden of Communicable Diseases in Europe (BCoDE) project by the European Center for Disease Prevention and Control and a related study on the infectious disease burden in the Netherlands (Colzani et al, 2017; Bijkerk et al. 2014; Kretzschmar et al. 2012; Mangen et al. 2013; van Lier et al. 2016).

The pathogen-based Markov disease models, including transition probabilities between and durations of health states, for chlamydia and gonorrhea were adopted from the most updated BCoDE toolkit (European Centre for Disease Prevention and Control 2020). The most updated GBD disability weights for each sequelae were substituted into the disease models in place of disability weights used in the BCoDE, which were elicited from a European population (James et al, 2018). For trichomoniasis, bacterial vaginosis, and candidiasis, no validated disease model outlining all long-term sequelae, transition probabilities, and durations could be found. Therefore, DALYs per trichomoniasis, bacterial vaginosis, and candidiasis case were estimated by considering those infections’ resultant increase in the risk of chlamydia, gonorrhea, and HIV (Brotman et al, 2010; Mavedzenge et al. 2010; van de Wijgert et al. 2008). However this method still overlooks other potential negative effects, such as pregnancy and birth outcomes or effects on HIV transmission to other individuals (Dingens et al. 2016; Cohen et al. 2012). The DALY estimates for trichomoniasis, bacterial vaginosis, and candidiasis also do not include the burden of the initial acute infections, which are negligible compared to the current estimate and do not have clear parameters in the existing literature.

III. Chlamydia (*C. trachomatis*) health impact calculation


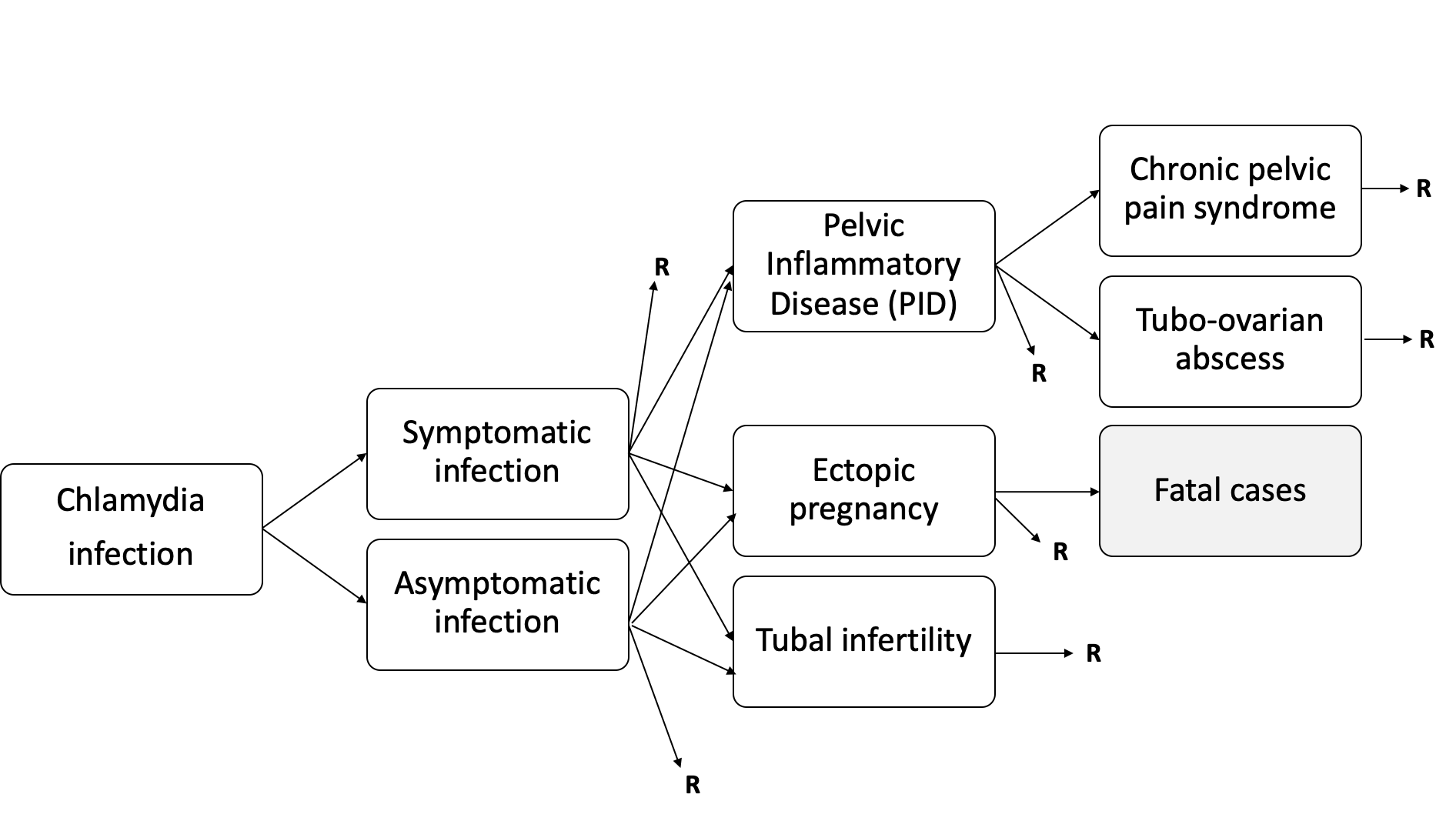


**Figure S1: Pathogen-based disease model for chlamydia. Adopted from Burden of Communicable Diseases in Europe, BCoDE v2.0.0 (European Centre for Disease Prevention and Control 2020).**

**Table S2: Inputs used in calculating DALYs per chlamydia infection and associated long-term sequelae.**


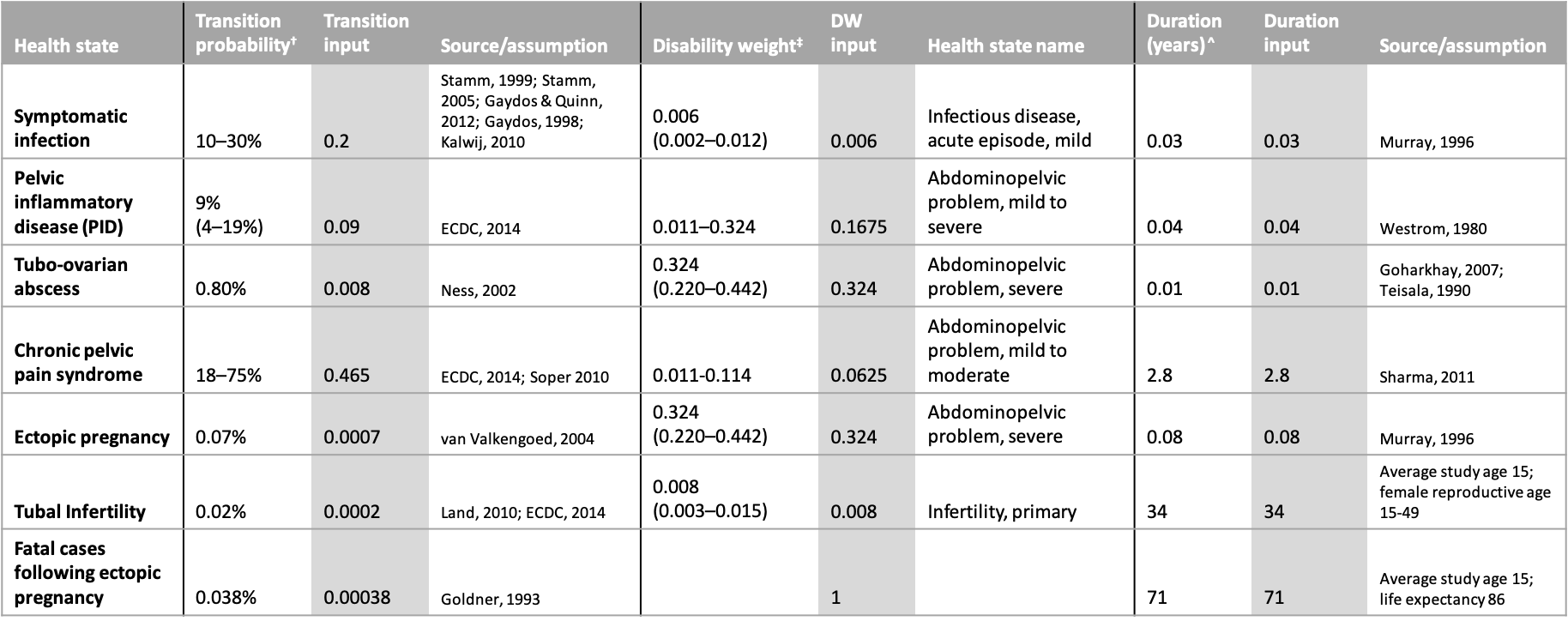


Gray columns indicate point estimates used as inputs in DALY calculation.

†Estimate reflects transition probability to health system from previous health state, not from initial infection. Compiled by BCoDE v2.0.0 (European Centre for Disease Prevention and Control 2020).

‡From Global Burden of Disease 2017 (James et al. 2018)

^Compiled by BCoDE v2.0.0 (European Centre for Disease Prevention and Control 2020).

**Table S3: DALY calculation per chlamydia infection and associated long-term sequelae.**


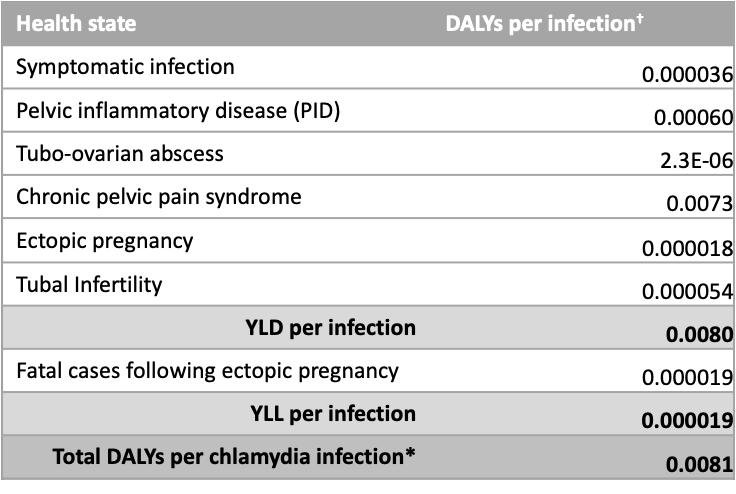


†Calculated using disability weight, duration, and probability of respective health state.

*Infection is either symptomatic or asymptomatic case.

Based on information in Table S1.

IV. Gonorrhea (*N. gonorrhea*) health impact calculation


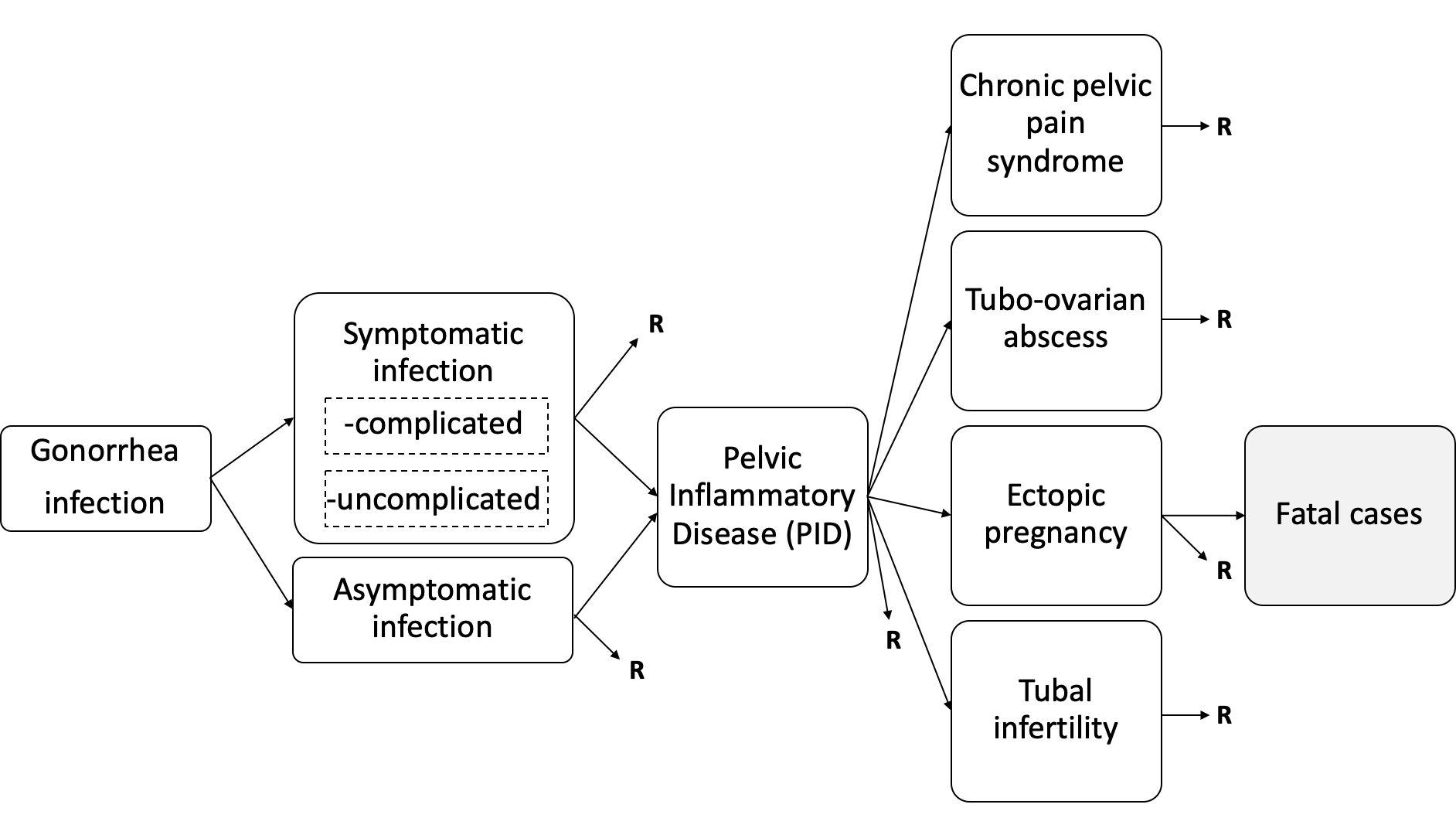


**Figure S2: Pathogen-based disease model for gonorrhea. Adopted from Burden of Communicable Diseases in Europe, BCoDE v2.0.0 (European Centre for Disease Prevention and Control 2020).**

**Table S4: Inputs used in calculating DALYs per gonorrhea infection and associated long-term sequelae.**


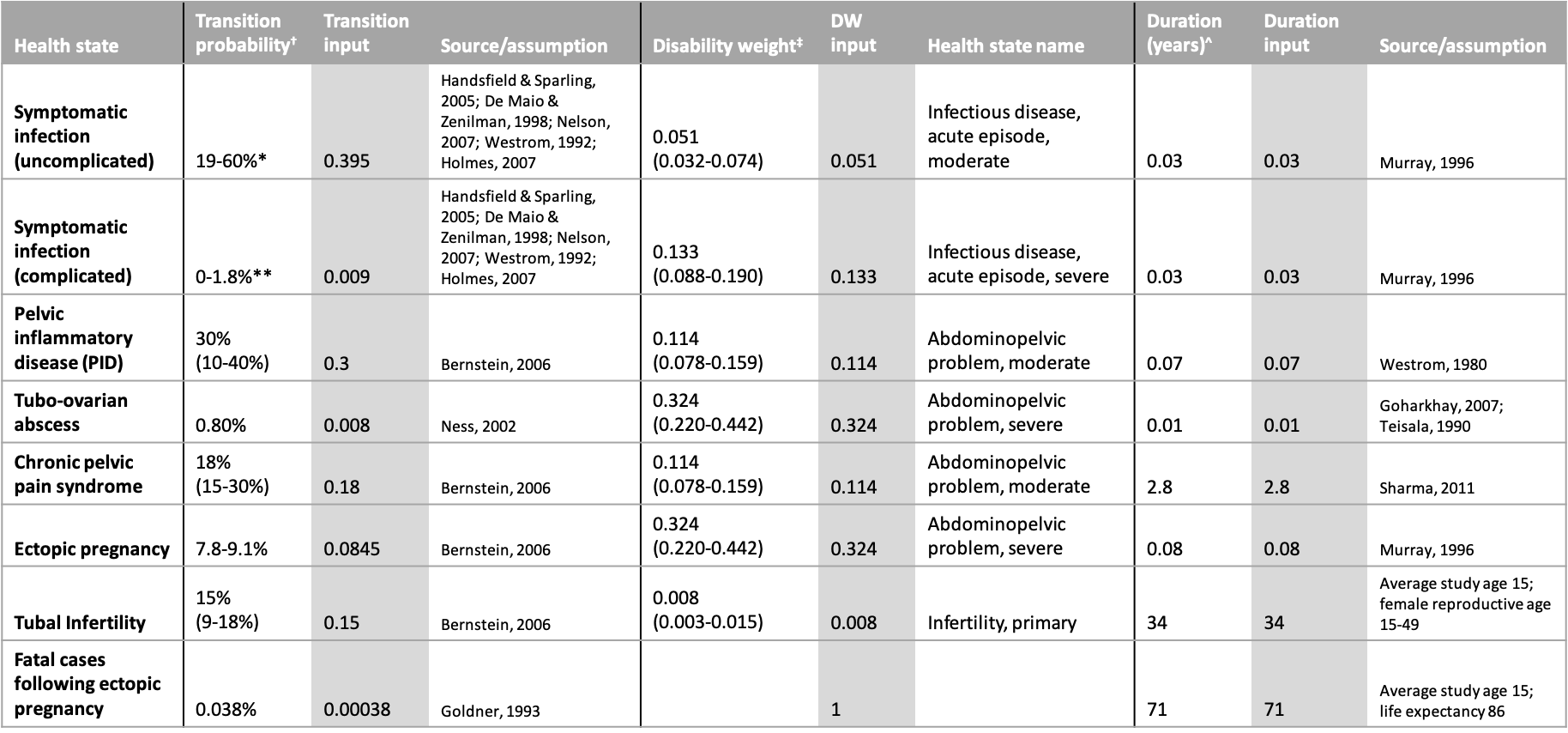


Gray columns indicate point estimates used as inputs in DALY calculation.

†Estimate reflects transition probability to health system from previous health state, not from initial infection. Compiled by BCoDE v2.0.0 (European Centre for Disease Prevention and Control 2020).

‡From Global Burden of Disease 2017 (James et al. 2018)

^Compiled by BCoDE v2.0.0 (European Centre for Disease Prevention and Control 2020).

*20-60% transition probability to overall symptomatic infection, and 97-99.5% of symptomatic infections are uncomplicated, resulting in specific transition probability of 19-60% to uncomplicated symptomatic infections.

**20-60% transition probability to overall symptomatic infection, and 0.5-3% of symptomatic infections are complicated, resulting in specific transition probability of 0-1.8% to complicated symptomatic infections.

**Table S5: DALY calculation per gonorrhea infection and associated long-term sequelae.**


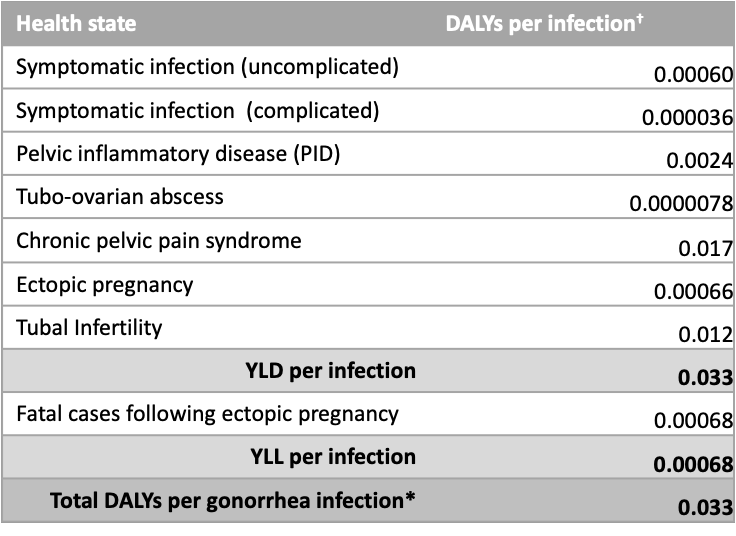


†Calculated using disability weight, duration, and probability of respective health state.

*Infection is either symptomatic or asymptomatic case.

Based on information in Table S3.

V. HIV health impact calculation (for use in health impact calculation for other infections)


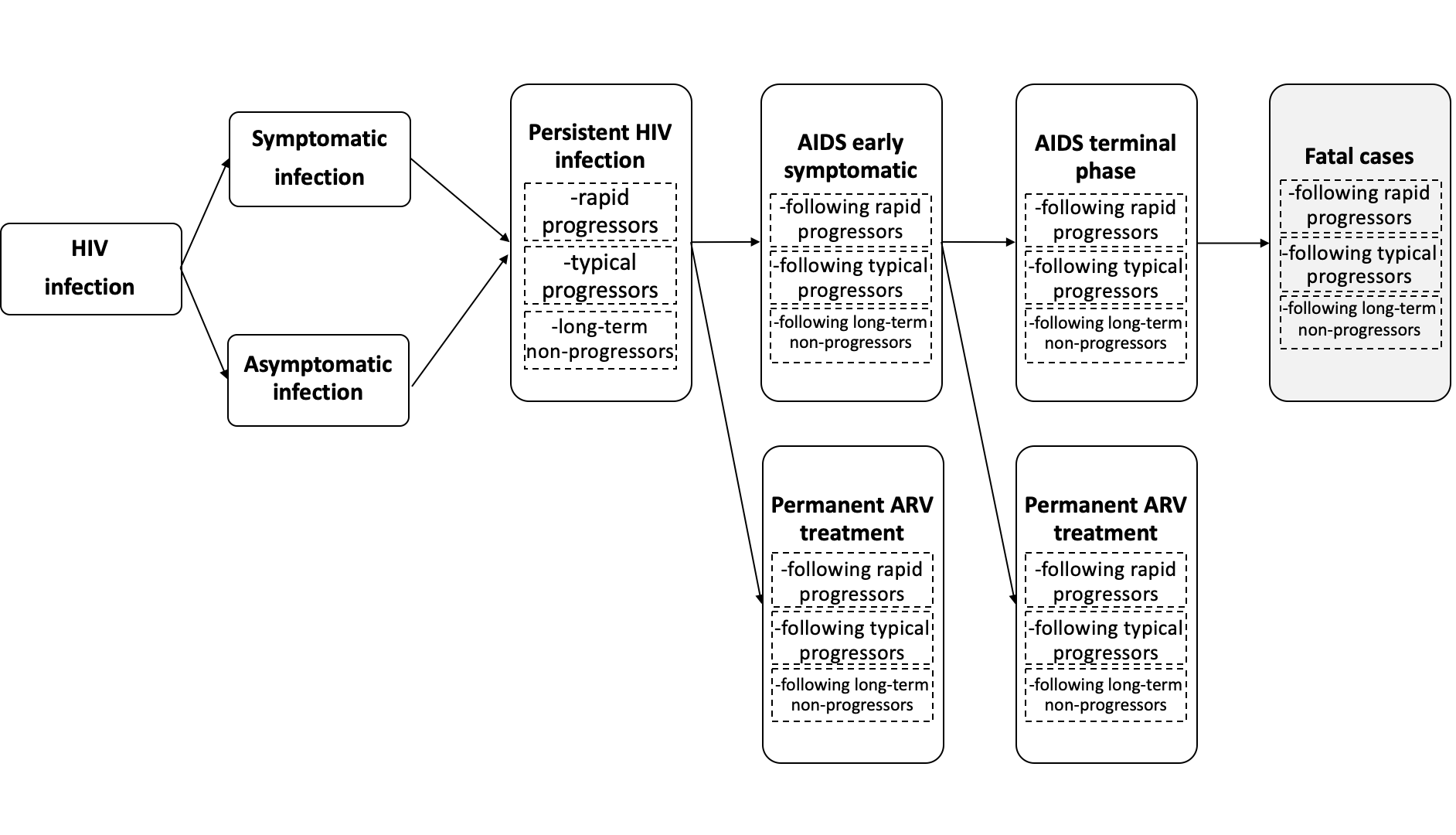


**Figure S3: Pathogen-based disease model for HIV. Adopted from Burden of Communicable Diseases in Europe, BCoDE v2.0.0 (European Centre for Disease Prevention and Control 2020).**

**Table S6: Inputs used in calculating DALYs per HIV infection and associated long-term sequelae.**


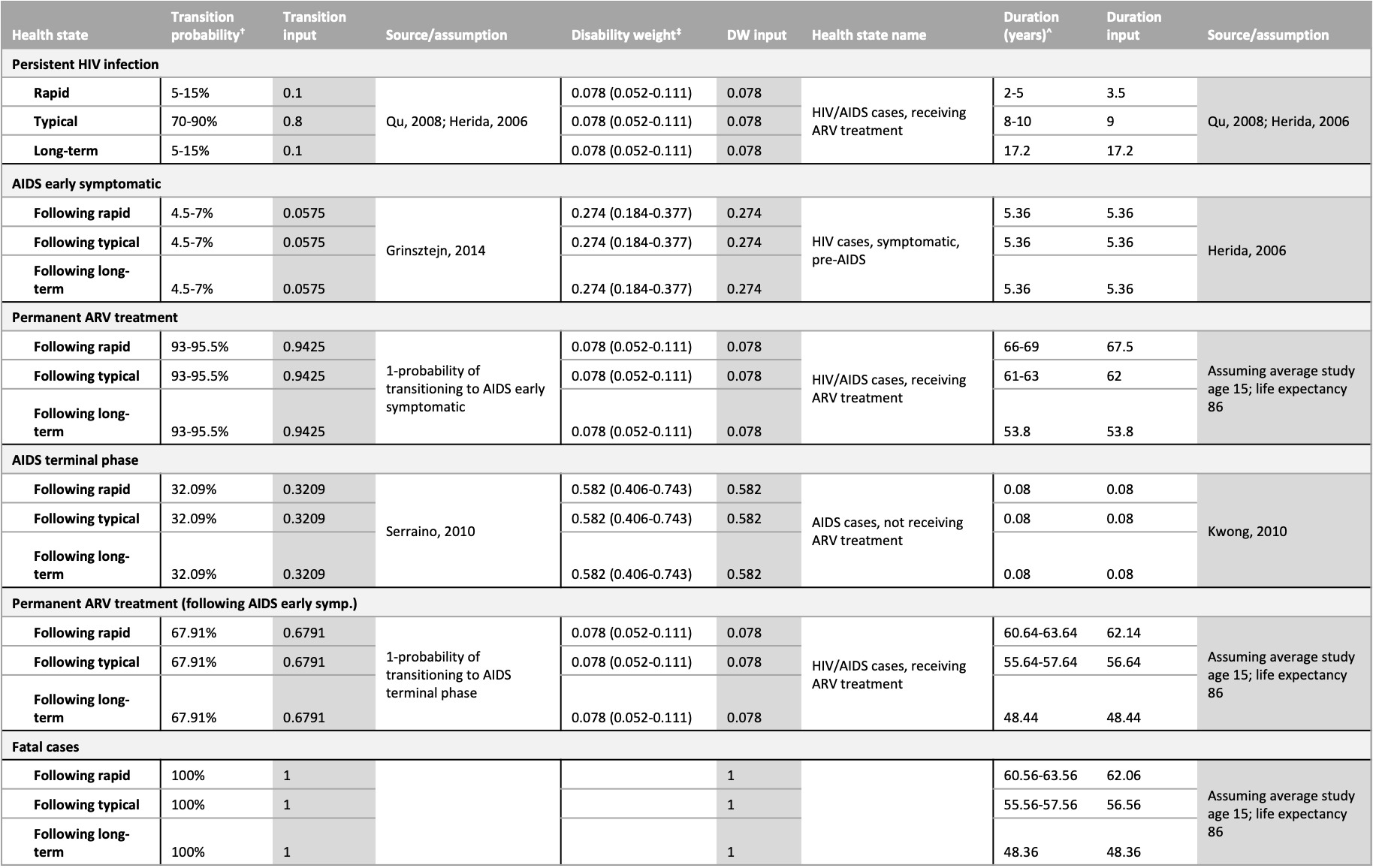


Gray columns indicate point estimates used as inputs in DALY calculation.

†Estimate reflects transition probability to health system from previous health state, not from initial infection. Compiled by BCoDE v2.0.0 (European Centre for Disease Prevention and Control 2020).

‡From Global Burden of Disease 2017 (James et al. 2018)

^Compiled by BCoDE v2.0.0 (European Centre for Disease Prevention and Control 2020).

**Table S7: DALY calculation per HIV infection and associated long-term sequelae.**


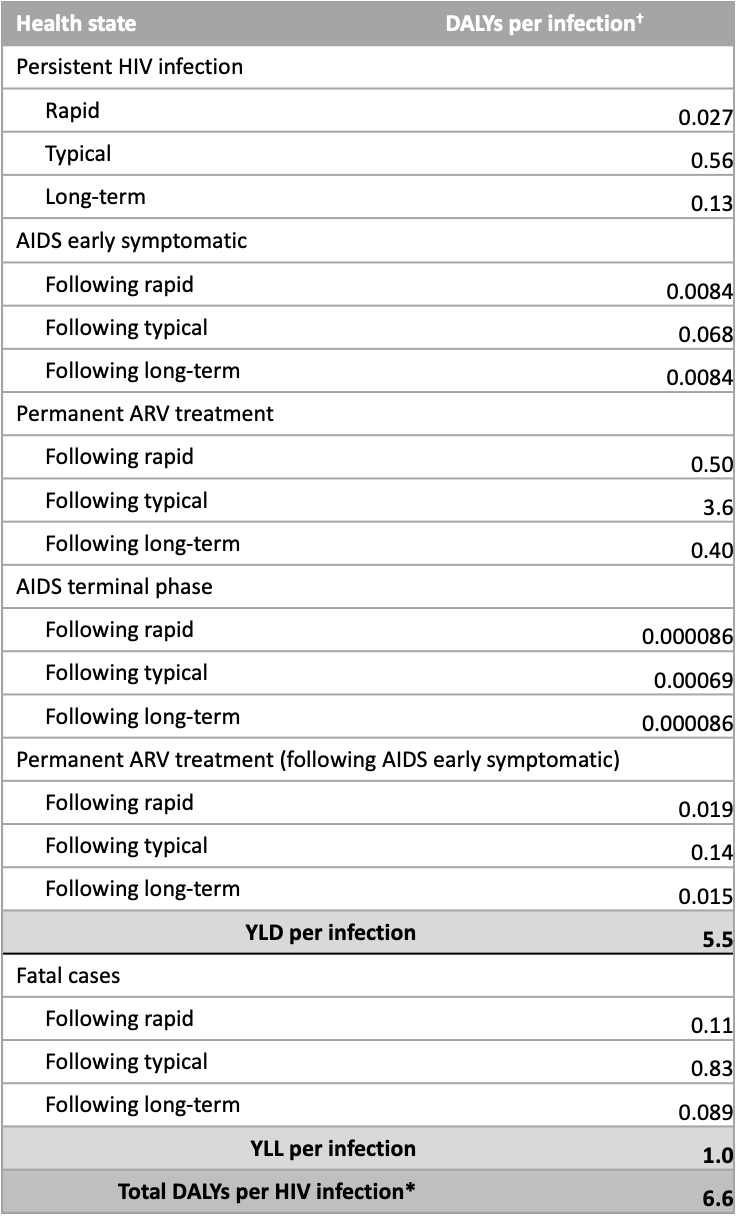


†Calculated using disability weight, duration, and probability of respective health state.

*Infection is either symptomatic or asymptomatic case.

Based on information in Table S5.

VI. Bacterial vaginosis (BV) health impact calculation

**Table S8: DALY calculation per bacterial vaginosis infection as a result of increased risk for other health states.**


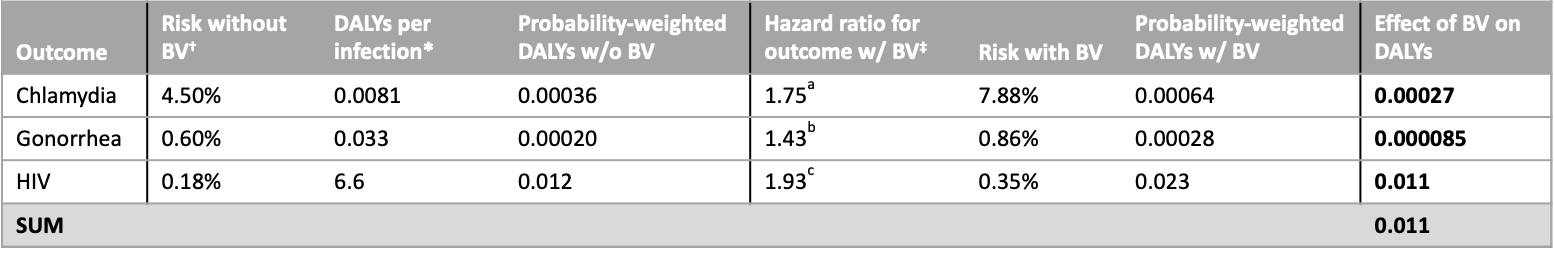


† Baseline risk of chlamydia and gonorrhea in this population estimated by prevalence of each outcome in control group of RCT (Phillips-Howard et al. 2016). Baseline risk of HIV in this population estimated by incidence of HIV in Kenya in 2017 (National AIDS Control Council 2018).

* Calculated considering all long-term sequelae for each outcome based on BCoDE models.

‡ Hazard ratio of impact of bacterial vaginosis on subsequent risk for each outcome.

^a^ Hazard ratio of incident chlamydia given bacterial vaginosis (7-10 Nugent Gram stain score) at visit 3 months prior. Adjusted for age, ethnicity, number of sex partners, condom use, other medications, vaginal douching, and other STI at prior visit (Brotman et al. 2010).

^b^ Hazard ratio of incident gonorrhea given bacterial vaginosis (7-10 Nugent Gram stain score) at visit 3 months prior. Adjusted for age, ethnicity, number of sex partners, condom use, other medications, vaginal douching, and other STI at prior visit (Brotman et al. 2010).

^c^ Hazard ratio of incident HIV given bacterial vaginosis (7-10 Nugent Gram stain score) at preceding visit within 6 months (van de Wijgert et al. 2008). Adjusted for country.

VII. Trichomoniasis (*T. vaginalis*) health impact calculation

**Table S9: DALY calculation per trichomoniasis infection as a result of increased risk for other health states.**


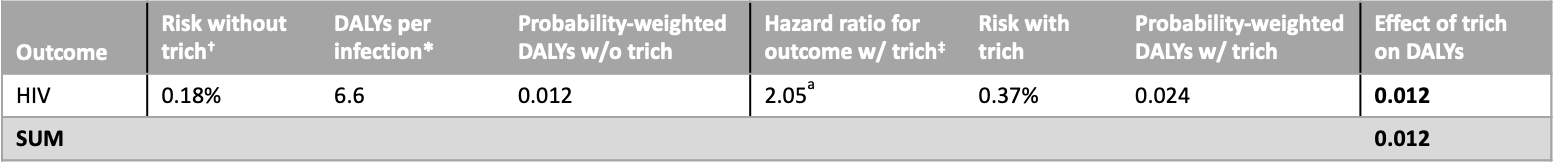


† Baseline risk of HIV in this population estimated by incidence of HIV in Kenya in 2017 (Naional AIDS Control Council 2018).

* Calculated considering all long-term sequelae for each outcome based on BCoDE models.

‡ Hazard ratio of impact of trichomoniasis on subsequent risk for each outcome.

^a^ Hazard ratio of incident HIV given trichomoniasis at preceding visit 3 months prior (Mavedzenge et al. 2010). Adjusted for age, education, living with partner, condom use at last sex, participant behavioral risk, primary partner behavioral risk, hormonal contraception, and other STIs.

VIII. *C. albicans* infection health impact calculation

**Table S10: DALY calculation per trichomoniasis infection as a result of increased risk for other health states.**


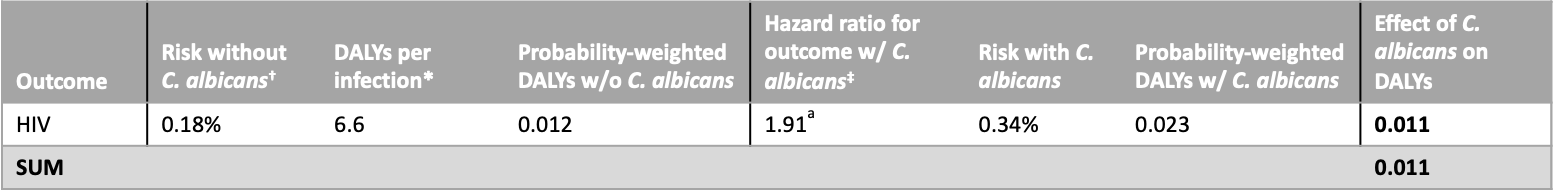


† Baseline risk of HIV in this population estimated by incidence of HIV in Kenya in 2017 (National AIDS Control Council 2018).

* Calculated considering all long-term sequelae for each outcome based on BCoDE models.

‡ Hazard ratio of impact of C. albicans infection on subsequent risk for each outcome.

^a^ Hazard ratio of incident HIV given candidiasis (including but not limited to C. albicans) at preceding visit within 6 months (van de Wijgert et al, 2008). Adjusted for country.

IX. Costs and outcomes overview

**Table S11: Factors to consider in CB and CE analysis of menstrual hygiene interventions**

| Costs | Outcomes |
| --- | --- |
| Material costs (cloth, pads, menstrual cup)  Education and training  Soap and hygiene  Maintenance (cleaning costs, including firewood)  Environmental costs (disposal)  Social costs (stigma, visibility of method) | Sexually transmitted infections  Reproductive tract infections  School dropout  School absenteeism  School performance  Future wages and productivity  Psychosocial effects |

X. Trial flow diagram

**Figure S4: Flow diagram of study participants** (Reprint from Phillips-Howard et al, 2016)

XI. CEA/CBA sensitivity analyses based on range of product costs

**Table S12: Sensitivity analysis of program costs for each treatment arm of study. Costs are considered from the perspective of a government/healthcare program, excluding private costs. Values in brackets indicate range considered for sensitivity analysis.**

|  | Treatment Arm | (1) Control^‡^ | | (2) Menstrual Cups | | (3) Sanitary Pads | |
| --- | --- | --- | --- | --- | --- | --- | --- |
|  | Item | *Annual cost per student (USD)* | Notes/assumptions | *Annual cost per student (USD)* | Notes/assumptions | *Annual cost per student (USD)* | Notes/assumptions |
| Material costs | Sanitary pads |  |  |  |  | 24  [12, 36] | 1 USD/pack, range 0.50-1.50 USD; 2packs/month |
|  | Menstrual cups |  |  | 1.00  [0.80, 4.2] | Total cost 10 USD, range 8-42 USD (van Eijk et al. 2019); single cup lasts 10 years |  |  |
|  | Replacement for lost menstrual cups |  |  | 0.05 | 6.3% of students lost their menstrual cups (van Eijk et al. 2018) |  |  |
| Menstrual cup training | Repeat training for students by nurses |  |  | 1.53 | Two half-day class repeat trainings required annually; 22 students on average per class |  |  |
|  | Training for girls, menstrual cup usage |  |  | 0.19 | 1 hour of class training in addition to puberty education |  |  |
|  | Training materials, menstrual cup usage |  |  | 0.50 |  |  |  |
| Control costs (puberty education and hygiene) | Soap for hygiene | 1.50 | 1 soap required per term; total 3 terms per year | 1.50 | 1 soap required per term; total 3 terms per year | 1.50 | 1 soap required per term; total 3 terms per year |
|  | Training for nurses | 0.17 | 3 hours training required; each nurse trains 75 students on average | 0.17 | 3 hours training required; each nurse trains 75 students on average | 0.17 | 3 hours training required; each nurse trains 75 students on average |
|  | Training for girls, puberty education | 0.77 | 2 hours of class training plus travel time; 22 students on average per class | 0.77 | 2 hours of class training plus travel time; 22 students on average per class | 0.77 | 2 hours of class training plus travel time; 22 students on average per class |
|  | Training materials, puberty education | 1.00 |  | 1.00 |  | 1.00 |  |
|  |  | **TOTAL 3.44 USD** | | **TOTAL 6.71 USD [6.51, 9.91 USD]** | | **TOTAL 27.44 USD [15.44, 39.44 USD]** | |
|  |  |  | | **RELATIVE TO CONTROL 3.27 USD***  **[3.07, 6.47 USD]** | | **RELATIVE TO CONTROL 24.00 USD***  **[12.00, 36.00 USD]** | |

^‡^The private cost of usual practice varies based on individual practices.

^*^The cost of each intervention relative to the control arm was used in cost-effectiveness and cost-benefit analyses since the health and education effects of each intervention were also measured in comparison to the control arm.

**Table S13: Sensitivity analysis of costs and benefits (USD) for providing menstrual cups or sanitary pads to 1,000 school-age girls for one year.**

|  | (1) Relative program cost | (2) Relative health effects | | | | (3) Relative education effects | | |
| --- | --- | --- | --- | --- | --- | --- | --- | --- |
|  |  | (a) DALYs averted | (b) CEA (USD/DALY averted) | (c) Valuation of averted DALYs | (d) CBA | (a) CEA (USD/student-school year) | (b) Valuation of increased student-school years | (c) CBA |
| Menstrual cups  program (1000 individuals) | $3,270  [$3,070, $6,470] | 1.4  [-4.3, 3.1] | $2300/DALY averted ($1000, dominated) | $6900  (-$21000, $15000) | **Net: +$3630**  **(-$27470, +$11930)** | *No significant effects of menstrual cup provision on absenteeism* | *No significant effects of menstrual cup provision on absenteeism* | **Net: -$3270** |
| Sanitary pads program (1000 individuals) | $24,000  [$12000, $36000] | 0.48  [-4.2, 2.3] | $50000/DALY averted ($5200, dominated) | $2400  (-$21000, $11000) | **Net: -$21600**  **(-$57000,**  **-$1000)** | $300/student-school year ($100, dominated)^†^ | $92000  (-$8000, $193000) | **Net: +$68000 (-$44000, +181000)** |

Values in brackets indicate range considered for sensitivity analysis.

Values in parentheses calculated based on variability in relative program cost as well as either DALYs averted for health effects or student-school years gained for education effects.

^†^Based on education impacts of sanitary pad program reported by Benshaul-Tolonen et al.^10^

References

Bernstein, Kyle T., Supriya D. Mehta, Anne M. Rompalo, and Emily J. Erbelding. “Cost-Effectiveness of Screening Strategies for Gonorrhea among Females in Private Sector Care.” *Obstetrics and Gynecology* 107, no. 4 (April 2006): 813–21. <https://doi.org/10.1097/01.AOG.0000204187.86600.0a>.

Bijkerk, Paul, Alies van Lier, Scott A. McDonald, Jacco Wallinga, and Hester E. de Melker. “Appendix: State of Infectious Diseases in the Netherlands, 2013.” The Netherlands: National Institute for Public Health and the Environment, 2014. <https://www.rivm.nl/bibliotheek/rapporten/appendix150205001.pdf>.

Brotman, Rebecca M., Mark A. Klebanoff, Tonja R. Nansel, Kai F. Yu, William W. Andrews, Jun Zhang, and Jane R. Schwebke. “Bacterial Vaginosis Assessed by Gram Stain and Diminished Colonization Resistance to Incident Gonococcal, Chlamydial, and Trichomonal Genital Infection.” *The Journal of Infectious Diseases* 202, no. 12 (December 15, 2010): 1907–15. <https://doi.org/10.1086/657320>.

Cohen, Craig R., Jairam R. Lingappa, Jared M. Baeten, Musa O. Ngayo, Carol A. Spiegel, Ting Hong, Deborah Donnell, et al. “Bacterial Vaginosis Associated with Increased Risk of Female-to-Male HIV-1 Transmission: A Prospective Cohort Analysis among African Couples.” *PLoS Medicine* 9, no. 6 (2012): e1001251. <https://doi.org/10.1371/journal.pmed.1001251>.

Colzani, Edoardo, Alessandro Cassini, Daniel Lewandowski, Marie-Josee J. Mangen, Dietrich Plass, Scott A. McDonald, Alies van Lier, et al. “A Software Tool for Estimation of Burden of Infectious Diseases in Europe Using Incidence-Based Disability Adjusted Life Years.” *PLOS ONE* 12, no. 1 (January 20, 2017): e0170662. <https://doi.org/10.1371/journal.pone.0170662>.

DeMaio, J, and J Zenilman. “Gonococcal Infections.” In *Bacterial Infections of Humans: Epidemiology and Control*, edited by A Evans and P Brachman, 285–304. New York: Plenum Medical Book Company, 1998.

Devleesschauwer, Brecht, Arie H. Havelaar, Charline Maertens de Noordhout, Juanita A. Haagsma, Nicolas Praet, Pierre Dorny, Luc Duchateau, Paul R. Torgerson, Herman Van Oyen, and Niko Speybroeck. “Calculating Disability-Adjusted Life Years to Quantify Burden of Disease.” *International Journal of Public Health* 59, no. 3 (June 2014): 565–69. <https://doi.org/10.1007/s00038-014-0552-z>.

Dingens, Adam S., Tessa S. Fairfortune, Susan Reed, and Caroline Mitchell. “Bacterial Vaginosis and Adverse Outcomes among Full-Term Infants: A Cohort Study.” *BMC Pregnancy and Childbirth* 16, no. 1 (September 22, 2016): 278. <https://doi.org/10.1186/s12884-016-1073-y>.

European Centre for Disease Prevention and Control. *Burden of Communicable Diseases Toolkit* (version 2.0.0). Solna, 2020. <https://www.ecdc.europa.eu/en/publications-data/toolkit-application-calculate-dalys>.

———. “Chlamydia Control in Europe: Literature Review.” Stockholm: ECDC, 2014. <https://www.ecdc.europa.eu/en/publications-data/chlamydia-control-europe-literature-review>.

Gaydos, C. A., M. R. Howell, B. Pare, K. L. Clark, D. A. Ellis, R. M. Hendrix, J. C. Gaydos, K. T. McKee, and T. C. Quinn. “Chlamydia Trachomatis Infections in Female Military Recruits.” *The New England Journal of Medicine* 339, no. 11 (September 10, 1998): 739–44. <https://doi.org/10.1056/NEJM199809103391105>.

Gaydos, Charlotte A, and Thomas C Quinn. “Chlamydial Infections.” In *Harrison’s Principles of Internal Medicine*, edited by Dan L Longo, Anthony Fauci, Dennis L Kasper, Stephen L Hauser, J Larry Jameson, and Joseph Loscalzo, 18th Edition., 2:1421–32. New York: McGraw Hill, 2012.

Goharkhay, N., U. Verma, and F. Maggiorotto. “Comparison of CT- or Ultrasound-Guided Drainage with Concomitant Intravenous Antibiotics vs. Intravenous Antibiotics Alone in the Management of Tubo-Ovarian Abscesses.” *Ultrasound in Obstetrics & Gynecology: The Official Journal of the International Society of Ultrasound in Obstetrics and Gynecology* 29, no. 1 (January 2007): 65–69. <https://doi.org/10.1002/uog.3890>.

Goldner, T. E., H. W. Lawson, Z. Xia, and H. K. Atrash. “Surveillance for Ectopic Pregnancy--United States, 1970-1989.” *MMWR. CDC Surveillance Summaries: Morbidity and Mortality Weekly Report. CDC Surveillance Summaries* 42, no. 6 (December 17, 1993): 73–85.

Grinsztejn, Beatriz, Mina C Hosseinipour, Heather J Ribaudo, Susan Swindells, Joseph Eron, Ying Q Chen, Lei Wang, et al. “Effects of Early versus Delayed Initiation of Antiretroviral Treatment on Clinical Outcomes of HIV-1 Infection: Results from the Phase 3 HPTN 052 Randomised Controlled Trial.” *The Lancet. Infectious Diseases* 14, no. 4 (April 2014): 281–90. <https://doi.org/10.1016/S1473-3099(13)70692-3>.

Handsfield, HH, and P Frederick Sparling. “Neisseria Gonorrhoeae.” In *Principles and Practice of Infectious Diseases*, edited by Gerald L Mandell, John E Bennett, and Raphael Dolin, 6th Edition., 2:2514–29. Pennsylvania: Elsevier Inc, 2005.

Herida, Magid, Christine Larsen, Florence Lot, Anne Laporte, Jean-Claude Desenclos, and Françoise F. Hamers. “Cost-Effectiveness of HIV Post-Exposure Prophylaxis in France.” *AIDS* 20, no. 13 (August 22, 2006): 1753–61. <https://doi.org/10.1097/01.aids.0000242822.74624.5f>.

Holmes, King, P. Sparling, Walter Stamm, Peter Piot, Judith Wasserheit, Lawrence Corey, and Myron Cohen. *Sexually Transmitted Diseases, Fourth Edition*. 4 edition. New York: McGraw-Hill Professional, 2007.

James, Spencer L., Degu Abate, Kalkidan Hassen Abate, Solomon M. Abay, Cristiana Abbafati, Nooshin Abbasi, Hedayat Abbastabar, et al. “Global, Regional, and National Incidence, Prevalence, and Years Lived with Disability for 354 Diseases and Injuries for 195 Countries and Territories, 1990–2017: A Systematic Analysis for the Global Burden of Disease Study 2017.” *The Lancet* 392, no. 10159 (November 10, 2018): 1789–1858. <https://doi.org/10.1016/S0140-6736(18)32279-7>.

Kalwij, Sebastian, Mary Macintosh, and Paula Baraitser. “Screening and Treatment of Chlamydia Trachomatis Infections.” *BMJ (Clinical Research Ed.)* 340 (April 21, 2010): c1915. <https://doi.org/10.1136/bmj.c1915>.

Kretzschmar, Mirjam, Marie-Josée J. Mangen, Paulo Pinheiro, Beate Jahn, Eric M. Fèvre, Silvia Longhi, Taavi Lai, et al. “New Methodology for Estimating the Burden of Infectious Diseases in Europe.” *PLOS Medicine* 9, no. 4 (April 17, 2012): e1001205. <https://doi.org/10.1371/journal.pmed.1001205>.

Kwong, JC, NS Crowcroft, MA Campitelli, S Ratnasingham, N Daneman, SL Deeks, and DG Manuel. “Ontario Burden of Infectious Disease Study Advisory Group; Ontario Burden of Infectious Disease Study (ONBOIDS): An OAHPP/ICES Report.” Toronto: Ontario Agency for Health Protection and Promotion, Institute for Clinical Evaluative Sciences, 2010.

Land, J. A., J. E. a. M. Van Bergen, S. A. Morré, and M. J. Postma. “Epidemiology of Chlamydia Trachomatis Infection in Women and the Cost-Effectiveness of Screening.” *Human Reproduction Update* 16, no. 2 (April 2010): 189–204. <https://doi.org/10.1093/humupd/dmp035>.

Lier, Alies van, Scott A. McDonald, Martijn Bouwknegt, Mirjam E. Kretzschmar, Arie H. Havelaar, Marie-Josée J. Mangen, Jacco Wallinga, and Hester E. de Melker. “Disease Burden of 32 Infectious Diseases in the Netherlands, 2007-2011.” *PLoS ONE* 11, no. 4 (April 20, 2016). <https://doi.org/10.1371/journal.pone.0153106>.

Mangen, Marie-Josée J., Dietrich Plass, Arie H. Havelaar, Cheryl L. Gibbons, Alessandro Cassini, Nikolai Mühlberger, Alies van Lier, et al. “The Pathogen- and Incidence-Based DALY Approach: An Appropriated Methodology for Estimating the Burden of Infectious Diseases.” *PLOS ONE* 8, no. 11 (November 20, 2013): e79740. <https://doi.org/10.1371/journal.pone.0079740>.

Mavedzenge, Sue Napierala, Barbara Van Der Pol, Helen Cheng, Elizabeth T. Montgomery, Kelly Blanchard, Guy de Bruyn, Gita Ramjee, and Ariane van der Straten. “Epidemiological Synergy of Trichomonas Vaginalis and HIV in Zimbabwean and South African Women.” *Sexually Transmitted Diseases* 37, no. 7 (July 2010): 460–66. <https://doi.org/10.1097/OLQ.0b013e3181cfcc4b>.

Murray, Christopher J. L., and Alan D. Lopez. *The Global Burden of Disease : A Comprehensive Assessment of Mortality and Disability from Diseases, Injuries, and Risk Factors in 1990 and Projected to 2020 : Summary*. Harvard School of Public Health on behalf of the World Health Organization and the World Bank, 1996. <https://apps.who.int/iris/handle/10665/41864>.

National AIDS Control Council. “Kenya HIV Estimates: Report 2018.” Kenya Ministry of Health, October 2018. <https://nacc.or.ke/wp-content/uploads/2018/11/HIV-estimates-report-Kenya-20182.pdf>.

Nelson, A L, A L Nelson, J Woodward, and S Wysocki. “Gonorrheal Infections.” In *Sexually Transmitted Infections - Current Clinical Practice*, 153–82. Humana Press, 2007.

Ness, Roberta B., David E. Soper, Robert L. Holley, Jeffrey Peipert, Hugh Randall, Richard L. Sweet, Steven J. Sondheimer, et al. “Effectiveness of Inpatient and Outpatient Treatment Strategies for Women with Pelvic Inflammatory Disease: Results from the Pelvic Inflammatory Disease Evaluation and Clinical Health (PEACH) Randomized Trial.” *American Journal of Obstetrics and Gynecology* 186, no. 5 (May 2002): 929–37. <https://doi.org/10.1067/mob.2002.121625>.

Phillips-Howard, Penelope A., Elizabeth Nyothach, Feiko O. ter Kuile, Jackton Omoto, Duolao Wang, Clement Zeh, Clayton Onyango, et al. “Menstrual Cups and Sanitary Pads to Reduce School Attrition, and Sexually Transmitted and Reproductive Tract Infections: A Cluster Randomised Controlled Feasibility Study in Rural Western Kenya.” *BMJ Open* 6, no. 11 (November 1, 2016): e013229. <https://doi.org/10.1136/bmjopen-2016-013229>.

Qu, Wei, Matthew Robinson, and Fu-jie Zhang. “Factors Influencing the Natural History of HIV-1 Infection.” *Chinese Medical Journal* 121, no. 24 (December 20, 2008): 2613–21.

Serraino, Diego, Silvia Bruzzone, Antonella Zucchetto, Barbara Suligoi, Angela De Paoli, Simona Pennazza, Laura Camoni, Luigino Dal Maso, Paoli De Paoli, and Giovanni Rezza. “Elevated Risks of Death for Diabetes Mellitus and Cardiovascular Diseases in Italian AIDS Cases.” *AIDS Research and Therapy* 7, no. 1 (May 24, 2010): 11. <https://doi.org/10.1186/1742-6405-7-11>.

Sharma, Damyanti, Krishna Dahiya, Nirmala Duhan, and Ruchi Bansal. “Diagnostic Laparoscopy in Chronic Pelvic Pain.” *Archives of Gynecology and Obstetrics* 283, no. 2 (February 2011): 295–97. <https://doi.org/10.1007/s00404-010-1354-z>.

Soper, David E. “Pelvic Inflammatory Disease.” *Obstetrics and Gynecology* 116, no. 2 Pt 1 (August 2010): 419–28. <https://doi.org/10.1097/AOG.0b013e3181e92c54>.

Stamm, Walter E. “Chlamydia Trachomatis Infections of the Adult.” In *Sexually Transmitted Diseases*, edited by King Holmes, P Frederick Sparling, and Per-Anders Mårdh, 3rd Edition., 407–22. New York: McGraw Hill, 1999.

Stamm, Walter E, Robert B Jones, and Byron E Battieger. “Chlamydia Trachomatis (Trachoma, Perinatal Infections, Lymphogranuloma Venerum and Other Genital Infections).” In *Principles and Practice of Infectious Diseases*, edited by Gerald L Mandell, John E Bennett, and Raphael Dolin, 6th Edition., 2:2239–55. Pennsylvania: Elsevier Inc, 2005.

Teisala, K., P. K. Heinonen, and R. Punnonen. “Transvaginal Ultrasound in the Diagnosis and Treatment of Tubo-Ovarian Abscess.” *British Journal of Obstetrics and Gynaecology* 97, no. 2 (February 1990): 178–80. <https://doi.org/10.1111/j.1471-0528.1990.tb01745.x>.

Valkengoed, Irene G. M. van, Servaas A. Morré, Adriaan J. C. van den Brule, Chris J. L. M. Meijer, Lex M. Bouter, and A. Joan P. Boeke. “Overestimation of Complication Rates in Evaluations of Chlamydia Trachomatis Screening Programmes--Implications for Cost-Effectiveness Analyses.” *International Journal of Epidemiology* 33, no. 2 (April 2004): 416–25. <https://doi.org/10.1093/ije/dyh029>.

Weström, L. “Incidence, Prevalence, and Trends of Acute Pelvic Inflammatory Disease and Its Consequences in Industrialized Countries.” *American Journal of Obstetrics and Gynecology* 138, no. 7 Pt 2 (December 1, 1980): 880–92. <https://doi.org/10.1016/0002-9378(80)91077-7>.

Weström, L., R. Joesoef, G. Reynolds, A. Hagdu, and S. E. Thompson. “Pelvic Inflammatory Disease and Fertility. A Cohort Study of 1,844 Women with Laparoscopically Verified Disease and 657 Control Women with Normal Laparoscopic Results.” *Sexually Transmitted Diseases* 19, no. 4 (August 1992): 185–92.

Wijgert, Janneke H. H. M. van de, Charles S. Morrison, Peter G. A. Cornelisse, Marshall Munjoma, Jeanne Moncada, Peter Awio, Jing Wang, et al. “Bacterial Vaginosis and Vaginal Yeast, but Not Vaginal Cleansing, Increase HIV-1 Acquisition in African Women.” *Journal of Acquired Immune Deficiency Syndromes* 48, no. 2 (June 1, 2008): 203–10. <https://doi.org/10.1097/QAI.0b013e3181743936>.
